# In Vitro Low-Bortezomib Doses Induce Apoptosis and Independently Decrease the Activities of Glutathione S-Transferase and Glutathione Peroxidase in Multiple Myeloma, Taking into Account the *GSTT1* and *GSTM1* Gene Variants

**DOI:** 10.3390/genes15030387

**Published:** 2024-03-21

**Authors:** Szymon Zmorzynski, Sylwia Popek-Marciniec, Beata Biernacka, Aneta Szudy-Szczyrek, Sylwia Chocholska, Wojciech Styk, Joanna Czerwik-Marcinkowska, Grazyna Swiderska-Kolacz

**Affiliations:** 1 Laboratory of Genetics, Academy of Zamosc, 22-400 Zamosc, Poland; 2 Institute of Nursing and Obstetrics, Academy of Zamosc, 22-400 Zamosc, Poland; 3 Chair and Department of Haematooncology and Bone Marrow Transplantation, Medical University of Lublin, 20-059 Lublin, Poland; anetaszudy@gmail.com (A.S.-S.); sylwiachocholska@umlub.pl (S.C.); 4 Academic Laboratory of Psychological Tests, Medical University of Lublin, 20-059 Lublin, Poland; wojciech.styk@gmail.com; 5 Institute of Biology, Jan Kochanowski University, 25-369 Kielce, Poland; marcinko@kielce.com.pl (J.C.-M.); grazyna.swiderska-kolacz@ujk.edu.pl (G.S.-K.)

**Keywords:** apoptosis, bortezomib, glutathione peroxidase, glutathione S-transferase, glutathione reductase, multiple myeloma, reduced glutathione

## Abstract

Background: Multiple myeloma (MM) is a malignancy derived from plasma cells. Bortezomib affects the concentration of reduced glutathione (GSH) and the activity of glutathione enzymes. The aim of our study was to analyze deletion (null/present) variants of *GSTT1* and *GSTM1* genes and their association with the levels of glutathione and its enzymes in bortezomib-treated cell cultures derived from MM patients. Materials and Methods: This study included 180 individuals (80 MM patients and 100 healthy blood donors) who were genotyped via multiplex PCR (for the *GSTT1*/*GSTM1* genes). Under in vitro conditions, MM bone marrow cells were treated with bortezomib (1–4 nM) to determine apoptosis (via fluorescence microscopy), GSH concentration, and activity of glutathione enzymes (via ELISA). Results: Bortezomib increased the number of apoptotic cells and decreased the activity of S-glutathione transferase (GST) and glutathione peroxidase (GPx). We found significant differences in GST activity between 1 nM (*GSTT1*-null vs. *GSTT1*-present), 2 nM (*GSTT1*-null vs. *GSTT1*-present), and 4 nM (*GSTM1*-null vs. *GSTM1*-present) bortezomib: 0.07 vs. 0.12, *p* = 0.02; 0.06 vs. 0.10, *p* = 0.02; and 0.03 vs. 0.08, *p* = 0.01, respectively. Conclusions: Bortezomib affects the activities of GST and GPx. GST activity was associated with *GSTT1* and *GSTM1* variants but only at some bortezomib doses.

## 1. Introduction

Multiple myeloma (MM) is a plasma cell malignancy of the bone marrow [1]. Different factors are involved in the etiology and pathogenesis of this disease, including genetic factors [2]. In the treatment of MM, agents with various mechanisms of action are used. One of these agents is bortezomib, which plays a role as a potent and reversible inhibitor of the 26S proteasome. It is a protein complex responsible for the degradation of intracellular proteins. Bortezomib-mediated inhibition of the proteasome leads to cancer cell apoptosis [3].

Moreover, bortezomib affects redox homeostasis in multiple myeloma cells [4]. It decreases the levels of intracellular reduced glutathione (GSH), which is an important endogenous antioxidant [4]. It protects the genome, proteins, and fats against the harmful effects of reactive oxygen species (ROS) and regulates metabolic processes and apoptosis [5,6]. ROS neutralization and cellular oxidative stress handling involve the glutathione system, which includes GSH and interacts with glutathione enzymes, such as glutathione peroxidase (GPx, EC 1.11.1.9), glutathione reductase (GR, EC 1.8.1.7), and glutathione S-transferases (GSTs, EC. 2.5.1.18), [7]. GPx reduces hydrogen peroxide to water, while GR converts oxidized glutathione (GSSG) into its reduced form [8]. These differences depend on the presence of two types of GSTs: GST theta-1, which is encoded by the *GSTT1* gene (*locus* 22q11.2), and GST mu-1, which is encoded by the *GSTM1* gene (*locus* 1p13.3) [9,10]. The variants of the *GSTT1* and *GSTM1* genes are examples of deletion polymorphisms. The null genotype means that the coding regions of both alleles at a single *locus* are deleted. The null variants are located in coding regions of the *GSTT1* and *GSTM1* genes and are associated with deletions of all exons and introns. However, noncoding regions of each gene, including the promoter, 5′-UTR and 3′-UTR, are present. Null genotypes result in a complete lack of corresponding enzyme activity [11].

Considering the above, we decided to analyze the relationship between GSH concentration and the activity of glutathione enzymes (GST, GPx, and GR) in combination with bortezomib in vitro (doses of 1 nM, 2 nM, and 4 nM) and its proapoptotic effect on multiple myeloma cells, taking into account the variants of the *GSTT1* and *GSTM1* genes. The hypotheses of the present research assumed that the activity of antioxidant enzymes and concentrations of GSH would depend on the concentration of bortezomib in the culture medium, and that a higher concentration of bortezomib would result in greater dynamics of changes in the activity of antioxidant enzymes. Moreover, the correlation between apoptosis and the levels of the studied antioxidant markers was analyzed. To our knowledge, such analyses with low-bortezomib doses have not been carried out in multiple myeloma patients.

## 2. Materials and Methods

### 2.1. Patients and Samples

This study included 180 individuals, with 80 newly diagnosed patients with MM and 100 healthy blood donors. From the MM patients, bone marrow aspirates and peripheral blood samples were collected. MM patients were hospitalized (between 2013–2020) at the Chair and Department of Haematooncology and Bone Marrow Transplantation, Medical University of Lublin.

Peripheral blood obtained from 100 healthy blood donors (50 males and 50 females) served as control samples. Their mean age was 34.4 years (range 18–61 years). Healthy blood donors were selected from the Regional Blood Donation and Blood Treatment Center in Kielce. 

The study obtained a positive opinion from the Bioethics Committee at Medical University of Lublin (no. KE-0254/165/2013 and no. KE-0254/337/2016) and at Jan Kochanowski University of Kielce (No. KB-41/2016), according to the ethical standards established by the Helsinki Declaration. The patients and healthy blood donors provided written informed consent.

Peripheral blood (from MM patients and healthy blood donors) was used to isolate DNA and to determine variants of the *GSTT1* and *GSTM1* genes.

**Figure 1:**
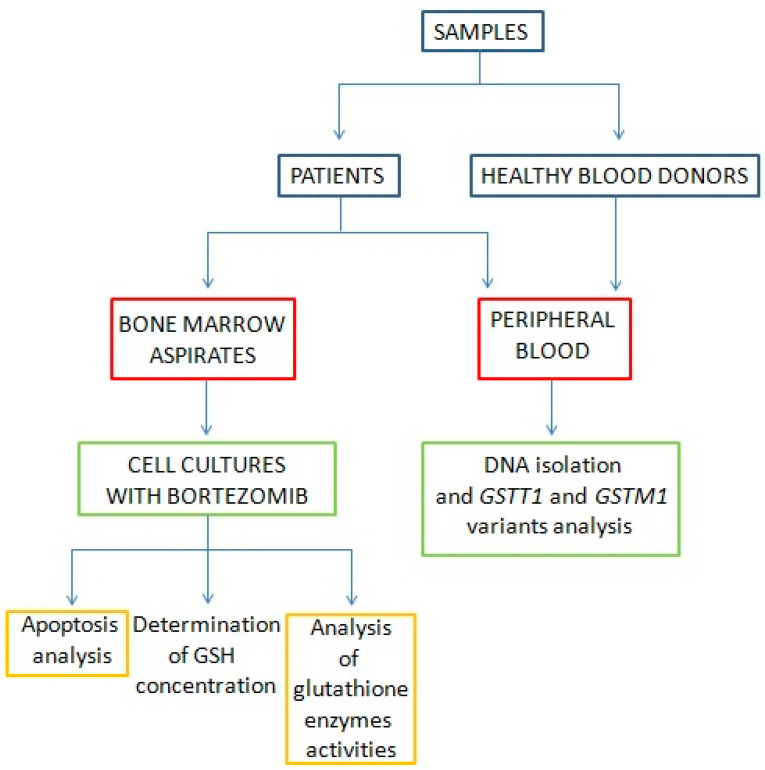
Experiment overflow.

Cell cultures were established from MM bone marrow aspirates to carry out in vitro research with bortezomib. Experiment overflow is shown in Figure 1.

The general characteristics of the MM patients are shown in Table 1.

### 2.2. Multiple Myeloma Cell Culture and In Vitro Bortezomib Treatment

Bone marrow aspirates were stratified on a Lymphoprep (Axis-Shield PoC As, Oslo, Norway), and the lymphocyte fraction was used to establish cell cultures, which were grown in 15 mL of culture medium (RPMI 1640) supplemented with L-glutamine (Biomed, Lublin, Poland), 10% inactivated fetal calf serum (Biomed, Lublin, Poland), 1% antibiotic antimycotic (A&E Scientific, Enghien, Belgium), and different doses (1 nM, 2 nM, or 4 nM) of bortezomib (LC Laboratories, Woburn, MA, USA, 200 mg/mL). Bortezomib was dissolved in DMSO (with its final concentration in the culture medium lower than 0.1%). The cell cultures without bortezomib (with 0.1% DMSO) were used as a control. Then, the lymphocyte fraction (about 1 mL) was added to the culture medium (with a volume of 15 mL). The cultures were carried out under appropriate conditions, at 37 °C and with 5% CO_2_ for 24 h (without granulocyte colony-stimulating factor) and were routinely terminated. The cell suspensions were used to determine the number of apoptotic/necrotic/viable cells, the GSH concentration, and the activities of GPx, GR, and GST.

### 2.3. In Vitro Determination of Apoptotic, Necrotic, and Viable Cells

**Figure 2:**
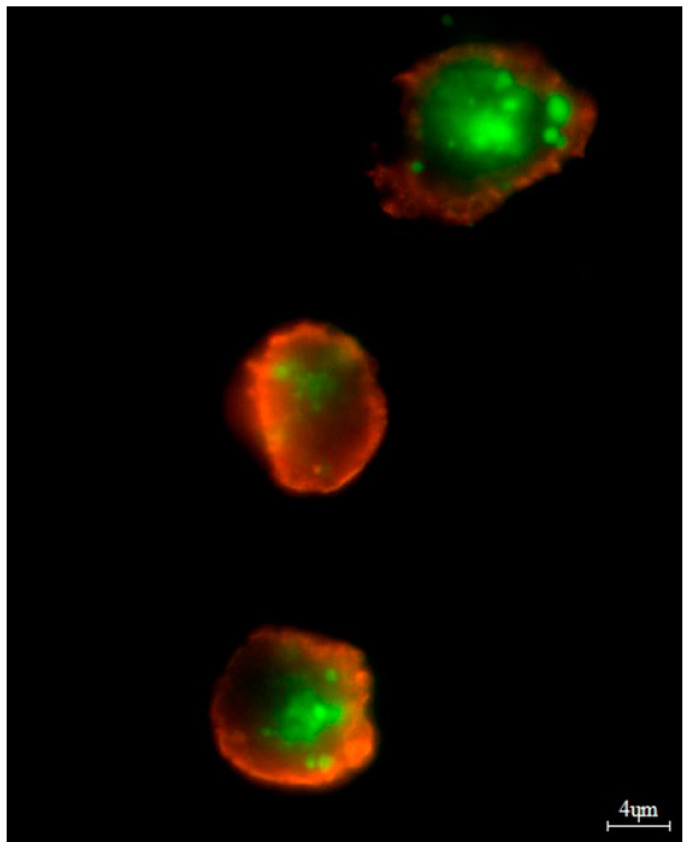
In vitro bortezomib treatment (at 4 nM). The apoptotic cells were stained with both AnnCy3 (red) and 6-CF (green). The cell at the top indicates early apoptosis. The cells in the middle and bottom panels indicate late apoptosis. For analysis, plasmocytes with a diameter of 9–12 μm were counted. The total magnification was 1500×. In this case, we observed: (I) 88.7% of viable cells, (II) 50.9% of apoptotic cells and 28% of necrotic cells.

Apoptotic, necrotic, and viable cells were detected with an Annexin V-Cy3 Apoptosis Detection Kit according to the manufacturer’s protocol (Millipore Sigma, Burlington, MA, USA). For fluorescence microscopy, viable cells were stained with 6-CF (6-carboxyfluorescein) (green), and necrotic cells were stained with AnnCy3 (Annexin V Cy3.18). Cells that had started the apoptotic process were stained with both AnnCy3 (red) and 6-CF (green) (Figure 2).

### 2.4. Trypan Blue Exclusion Test of Cell Viability

A volume of cell suspension with a volume of 0.4% trypan blue was mixed in a ratio of 1:1 and covered with a coverslip. After 3 min, changes in cell staining were observed, with the nuclei of dead cells stained blue. Cells were counted within 3 to 5 min of mixing with trypan blue.

### 2.5. Analysis of Glutathione Enzyme Activities

The activities of glutathione enzymes, GPx, GR, and GST were also analyzed.

#### 2.5.1. Glutathione Peroxidase (GPx) Activity

GPx activity was measured using a Glutathione Peroxidase Cellular Activity Assay Kit (Millipore Sigma, Burlington, MA, USA; cat. no. MAK437) according to the manufacturer’s instructions. Extinction was measured spectrophotometrically (TK Biotech, Warsaw, Poland) at a wavelength of λ = 340 nm every 15 s for 1 min. GPx activity was expressed in U· mg^−1^ protein.

#### 2.5.2. Glutathione Reductase (GR) Activity

GR activity was measured using a Glutathione Reductase Assay Kit (Millipore Sigma, Burlington, MA, USA; cat. no. GRSA). A plate reader (TK Biotech, Warsaw, Poland) was used to measure GR activity at a wavelength of 412 nm (in U · mg^−1^ protein).

#### 2.5.3. Glutathione S-transferase (GST) Activity

GST activity was measured using a Glutathione S-transferase (GST) Assay Kit (Millipore Sigma, Burlington, MA, USA; cat. no. MAK453). A plate reader (TK Biotech, Warsaw, Poland) was used to measure the GST activity at a wavelength of 340 nm (in U · mg^−1^ protein).

### 2.6. Determination of Reduced Glutathione (GSH) Concentration

The GSH concentration was determined using a Sigma Aldrich Glutathione Assay Kit (Millipore Sigma, Burlington, MA, USA; cat. no. MAK364) and a plate reader (TK Biotech, Warsaw, Poland) at a wavelength of 450 nm. The total protein concentration was determined according to the method of Lowry et al. [12].

### 2.7. DNA Isolation

DNA isolation from peripheral blood (from healthy blood donors, n = 100; from MM patients, n = 80) was performed using a commercial kit (Qiagen, Hilden, Germany) according to the manufacturer’s procedure. The concentration and quality of the DNA were checked using a NanoDrop device (Thermo Fisher Scientific, Waltham, MA, USA). DNA was used to determine the *GSTT1* and *GSTM1* gene variants via PCR.

### 2.8. Genotyping—Polymerase Chain Reaction (PCR) Multiplex

For analysis of *GSTT1* and *GSTM1* polymorphisms, the multiplex PCR method was applied. The β-globin gene was used as an internal control. The primers and band sizes obtained via multiplex PCR were determined as previously described [13].

For the multiplex PCR, the protocol described by Abdel-Rahman et al. was used with minor modifications [14].

**Figure 3:**
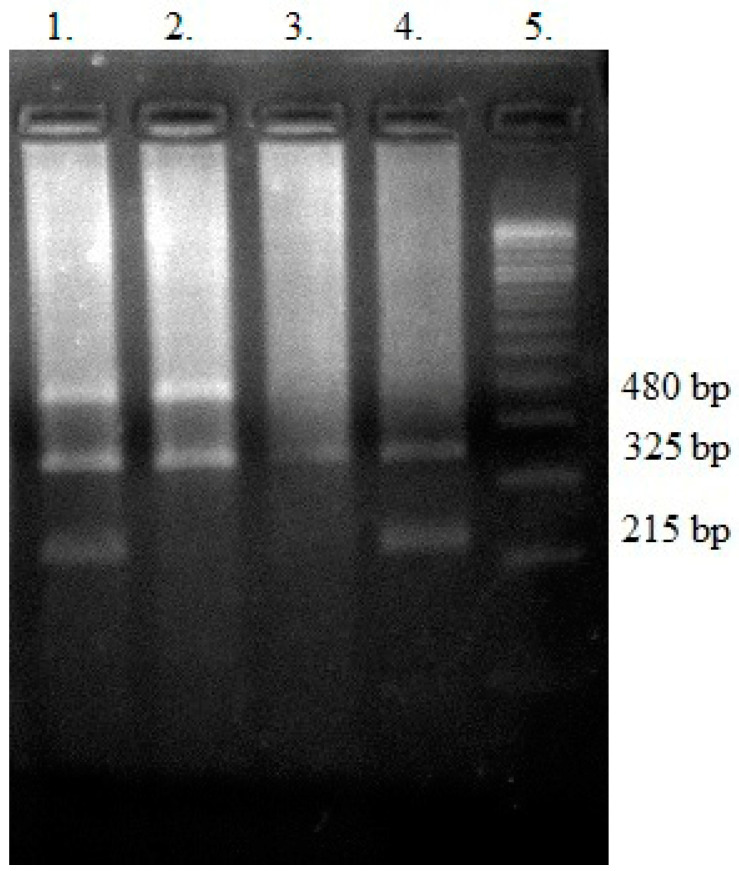
Electropherogram of *GSTT1* and *GSTM1* variants. Lane 1 contains bands 480 bp (for *GSTT1*-present), 215 bp (for *GSTM1*-present), and 325 bp (for the internal control). Lane 2 shows a 480 bp band (*GSTT1*-present) and an internal control (325 bp). The lack of 215 bp indicates the *GSTM1*-null genotype. Lane 3 contains only a band for internal control (325 bp), indicating *GSTT1*-null and *GSTM1*-null genotypes. Lane 4 shows bands 325 bp for internal control and 215 bp (for *GSTM1*-present). The lack of 480 bp indicates the *GSTT1*-null genotype. Lane 5 is a ladder (100 bp).

The PCR products were analyzed on 3% agarose gels, stained with SimplySafe (Eurx, Gdansk, Poland) and visualized in G:Box (Syngene, Cambridge, UK) (Figure 3). An independent PCR analysis was carried out for each sample.

### 2.9. Statistical Analysis

The laboratory values of MM patients with polymorphisms were compared using an independent *t* test for continuous variables and the chi-square test for categorical variables. The associations of the studied variants with prognostic factors were evaluated using the chi-square test or Fisher’s exact test (for expected values <5). The quantitative data are shown as the frequency or percentage. Deviation of genotype frequencies in controls and patients from Hardy-Weinberg equilibrium (HWE) was assessed by the chi-square test with Yates’s correction for the groups with <5 patients [15]. For the 95% confidence interval (CI), we assumed *p* = 0.05 and χ^2^ = 3.84; therefore, if χ^2^ ≤ 3.84 and the corresponding *p* ≥ 0.05, then the population was in HWE. Logistic regression was used to evaluate the fold change risk of MM. The Kaplan-Meier method and the log-rank test were used for survival analysis. We assumed a 5% error of inference, and a *p*-value < 0.05 indicated a statistically significant difference. Statistical analysis was performed using JASP 0.16.3 software.

## 3. Results

The present study included 180 individuals (80 MM patients and 100 healthy blood donors). The detailed clinical characteristics of the MM patients are shown in Table 1. In our in vitro study, bortezomib affected the number of viable, apoptotic, and necrotic cells (Table 2). Moreover, it changed the concentration of GSH, as well as glutathione enzymes.

### 3.1. Low Doses of Bortezomib Decreased the Number of Viable Cells and Induced Apoptosis in Multiple Myeloma

**Figure 4:**
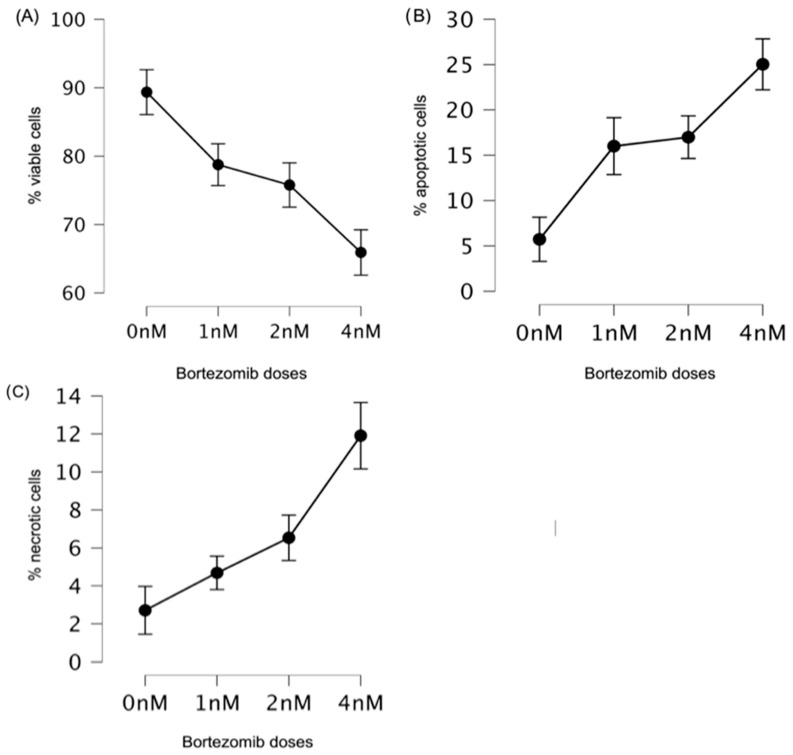
Effect of bortezomib on cell survival. In most cases, bortezomib significantly (**A**) decreased the number of viable cells, (**B**) increased the number of apoptotic cells, and (**C**) increased the number of necrotic cells at all studied doses (1–4 nM). As a control, samples without bortezomib (0 nM) were used. The graphs with mean values (in %) and with 95% confidence interval (95%CI) were shown.

The number of viable cells was assessed in a fluorescence microscopy test and in a trypan blue exclusion test of cell viability. We did not observe statistically significant results between results obtained in these two tests in control samples (89.36% ± 7.51% vs. 87.57% ± 6.88, *p* = 0.11), at 1 nM (78.75% ± 12.28% vs. 78.29% ± 14.17%, *p* = 0.82), at 2 nM (75.78% ± 12.64% vs. 76.69% ± 14.63%, *p* = 0.67), or at 4 nM of bortezomib (65.92% ± 12.78% vs. 69.94 ± 15.41%, *p* = 0.07, respectively). Considering that there were no significant differences in the two assays assessing cell viability, in further studies we included the number of viable cells assessed by fluorescence microscopy assay. Bortezomib significantly decreased the number of viable cells at all doses (1–4 nM) in comparison to the control (0 nM) (Figure 4A). 

Compared to those of the control (0 nM), the results obtained at all bortezomib doses (1–4 nM) were significantly different (Table 3). The differences between doses of 1 nM and 4 nM, as well as between 2 nM and 4 nM were significant (*p* < 0.01). The difference between 1 nM vs. 2 nM doses was statistically insignificant (Table 3).

Moreover, bortezomib increased the number of apoptotic and necrotic cells (Figure 4B,C). Compared to those of the control (0 nM) and between the studied bortezomib doses (except 1 nM vs. 2 nM), the differences in the number of apoptotic cells were statistically significant (Table 4).

For necrotic cells, similar results were observed to those obtained when analyzing the number of apoptotic cells. The percentage of necrotic cells was lower than that of apoptotic cells, and no statistically significant differences were observed between the 0 nM and 1 nM doses or between the 1 nM and 2 nM doses (Table 5).

### 3.2. Differences in the Reduced Glutathione (GSH) Concentration between the Control (0 nM) and Bortezomib Treatment Groups (1 nM, 2 nM, and 4 nM)

Bortezomib did not significantly change the level of GSH (Table 6). Moreover, a difference at the level of tendency was observed in the GSH concentration between the control (0 nM) and 4 nM of bortezomib (*p* = 0.05) (Table 6).

### 3.3. Changes in Glutathione Enzyme Activities

**Figure 5:**
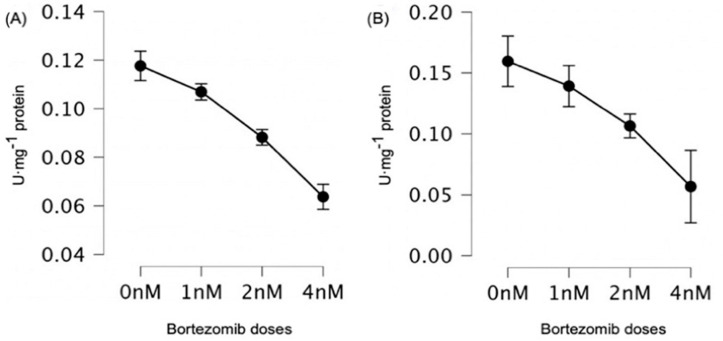
Differences in (**A**) GSH; and (**B**) GPx activities at all studied bortezomib doses (0–4 nM). As a control, samples without bortezomib (0 nM) were used. The graphs with mean values (in %) and with 95% confidence interval (95%CI) are shown.

Bortezomib significantly decreased GST activity at all bortezomib doses (Figure 5A, Table 7). For GR, the only significant difference was observed at a dose of 4 nM relative to the control (0 nM vs. 4 nM, *p* = 0.02) (Table 8). When analyzing the activity of GPx, we observed statistically significant differences between the control and 2 nM or 4 nM of bortezomib, as well as in the groups 1 nM vs. 4 nM and 2 nM vs. 4 nM (Table 9).

### 3.4. GSTT1 and GSTM1 Variants in the Context of the Analyzed Antioxidant Parameters

Genotyping was successful for all individuals investigated within the study. The HWE test confirmed that the genotypic frequencies (of *GSTT1* and *GSTM1*) for healthy individuals (controls) and MM patients were balanced (Table 10). The allelic frequencies of both *GST* variants between the study and control groups were not significantly different.

We did not observe an association between the *GSTT1* or *GSTM1* variant alone (Table 11) or in combination (Table 12) and the risk of MM. Moreover, these variants were not associated with the presence of chromosomal aberrations in MM patients; OR = 0.85 (0.22–3.08 95%CI), *p* = 0.94 for *GSTT1* variants, and OR = 0.36 (0.1–1.26 95%CI), *p* = 0.1 for *GSTM1* variants.

**Figure 6:**
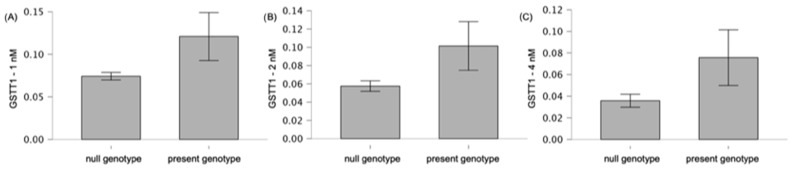
Differences in GST activity (U·mg^−1^ protein) at (**A**) 1 nM (*p* = 0.02), (**B**) 2 nM (*p* = 0.02), and (**C**) 4 nM (*p* = 0.08) of bortezomib, according to the type of *GSTT1* genotype (null vs. present). The graphs with mean values (in %) and with 95% confidence interval (95%CI) are shown.

We observed a statistically significant difference in the activity of GST at 1 nM and at 2 nM of bortezomib in the group with the null genotype of *GSTT1* gene and the present genotype of the *GSTT1* gene (null vs. present genotype); 0.07 vs. 0.12, *p* = 0.02 and 0.06 vs. 0.10, *p* = 0.02, respectively (Figure 6). In the case of 4 nM, a difference in GST activity (0.04 vs. 0.08 null genotype vs. present genotype, respectively) was observed at the level of tendency (*p* = 0.08).

**Figure 7:**
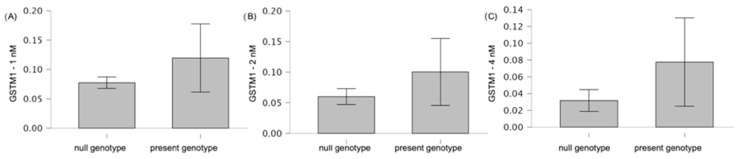
Differences in GST activity (U·mg^−1^ protein) at (**A**) 1 nM (*p* = 0.16), (**B**) 2 nM (*p* = 0.08), and (**C**) 4 nM (*p* = 0.01) of bortezomib, according to the type of *GSTM1* gene variant. The graphs with mean values (in %) and with 95% confidence interval (95%CI) are shown.

Taking into account the variant of the *GSTM1* gene (null genotype vs. present genotype), we found a statistically significant difference in GST activity at bortezomib doses of 4 nM (0.03 vs. 0.08, *p* = 0.01) (Figure 7). At the 2 nM dose, we observed a difference in GST activity between the null genotype and the present genotype of the *GSTM1* gene, with a trend of 0.06 vs. 0.10, *p* = 0.08.

**Figure 8:**
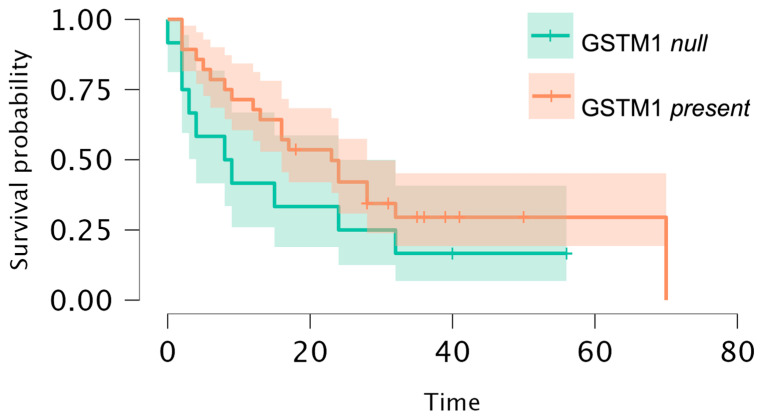
K-M analysis of PFS in the group of 80 MM patients with *GSTM1* genotype (log-rank test *p* = 0.05).

According to the log-rank test, we observed a difference in PFS at the level of tendency (*p* = 0.05) between the studied *GSTM1* variants (Figure 8). We did not observe a significant relationship between the levels of GSH or glutathione enzymes or between the number of apoptotic/necrotic/viable cells and OS or PFS.

### 3.5. Correlations of the Analyzed Antioxidant Parameters

To determine the associations between variables, we used Spearman’s partial correlation analysis to account for the additional effect of the bortezomib dose. We did not find correlations between the studied antioxidant parameters and the number of apoptotic/necrotic/viable cells. These findings indicate that the observed changes in GSH concentration and enzyme activity are not the result of apoptosis and/or necrosis.

We observed statistically significant correlations between GSH levels and GR activity (rho = 0.34, *p* < 0.001), between GSH levels and GPx activity (rho = 0.29, *p* < 0.001), between GST levels and GR activity (rho = −0.20, *p* < 0.05), and between GPx and GR activity (rho = 0.18, *p* < 0.05).

## 4. Discussion

In our study, we analyzed the concentration of GSH and the activities of glutathione enzymes (GST, GR, GPx) taking into account *GSTT1* and *GSTM1* variants. Glutathione and its enzymes are among the many indicators of oxidative stress. To our knowledge, this is the first study analyzing the glutathione concentration and enzyme activities in the context of deletion variants in the *GSTT1* and *GSTM1* genes and the response to bortezomib treatment (in low doses) in cell cultures derived from MM patients.

Medical drugs that affect the function of the ubiquitin-proteasome system have improved MM treatment efficacy [16]. Bortezomib, a proteasome inhibitor, leads to the accumulation of intracellular unfolded proteins and increases cellular stress [17]. MM patients exhibit increased systemic oxidative stress [18]. Bortezomib promotes cell death via multiple pathways, including overproduction of ROS, which are recognized as important secondary messengers involved in the regulation of cell signaling [19,20]. The generation of ROS induces the initiation of bortezomib-induced apoptosis [21]. Changes in the concentration and activity of cellular antioxidants are associated with increased susceptibility to bortezomib-induced apoptosis [22]. In our study, bortezomib significantly decreased the number of viable cells and increased the number of apoptotic and necrotic cells, which is consistent with the findings of other researchers [13,16]. To analyze the role of the antioxidant network in MM, cells derived from patients were treated with low doses of bortezomib at concentrations of 1 nM, 2 nM, and 4 nM, as these concentrations ranged from 1 to 100 nM within the first 24 h after in vivo bortezomib treatment [23].

Cancer cells frequently exhibit altered oxidative metabolism, resulting in intracellular oxidative stress [24]. Therefore, redox-directed therapies that inhibit the activity of antioxidant enzymes and decrease the concentration of GSH have been suggested to induce cytotoxicity in cancer cells, including malignant plasma cells [25,26]. Bortezomib-resistant cells exhibit increased GSH concentrations [27]. Nerini-Molteni et al. analyzed the relationships between redox homeostasis and bortezomib treatment in MM cells [4]. They found that decreasing intracellular GSH enhances bortezomib toxicity. A similar effect was observed by Starheim et al. [16]. Antioxidants protect MM cells from bortezomib-mediated cell death [16]. Moreover, depletion of GSH can enhance the effect of bortezomib in MM cells [4]. Cells with higher GSH levels are also more resistant to apoptosis [28]. A decrease in GSH levels impairs the antioxidant system and leads to an increase in ROS production [29]. The accumulation of ROS induces mitochondrial damage and apoptosis [29]. In turn, an increase in the GSH concentration is associated with cancer development [30]. Stimulation of GSH synthesis can inhibit apoptosis [31]. Hentze et al. reported that cancer cells have a greater GSH pool than normal tissue, which induces drug resistance [32]. In our study, we did not observe significant differences in GSH concentrations in samples treated with different bortezomib doses. This drug may increase the number of apoptotic cells, probably through a mechanism not associated with GSH levels. Furthermore, the dose of bortezomib used in the experiment may have been too low to disrupt glutathione homeostasis.

The antioxidant system includes a variety of enzymes, such as GSTs, GR, and GPx.

The GST enzymes play important roles in protecting genomic and cellular structures against ROS [9]. These enzymes eliminate toxic carcinogens [7,9]. In the present study, we observed a decrease in GST activity in cell cultures with increasing bortezomib doses. Moreover, taking into account the *GSTT1* and *GSTM1* variants, we found that, at some bortezomib doses, GST activity was lower in patients with null genotypes. In genes encoding GSTs, polymorphic changes in the form of deletions were described, with these variants affecting enzyme activity [33]. Deletion of *GST* alleles results in a lack of enzyme activity. This may increase the level of carcinogens and ROS, affecting the sequence of genes regulating cell cycle progression. In the case of *GST* present/null genotypes, we did not find an impact on MM risk, in contrast to our previous study on a larger MM patient group [13]. Moreover, in our previous study, individuals with the *GSTT1*-null or with both the *GSTT1*-null and *GSTM1*-null genotypes showed a greater risk for MM development than patients with non-deleted *GSTT1*/*GSTM1* genotypes. These findings are consistent with those obtained for hematological malignancies by other researchers [34,35,36]. Chen et al. found that the *GSTM1*-null genotype in combination with the *CYP1A1* and *CYP2D6* heterozygous mutant genotypes was associated with an elevated risk of acute non-lymphoblastic leukemia [11]. Yuille et al., in the study of *GSTM1, GSTT1,* and GSTP1 variants in chronic lymphocytic leukemia (CLL), observed an association between the *GSTM1*-null and *GSTT1*-null genotypes and the risk of CLL [37]. In the present study, we did not find an association between *GSTM1*/*GSTT1* variants and increased MM risk. This may be due to the low number of individuals included in the study.

In neuroblastoma cells, increased GPx activity promoted cytoprotection against proteasome inhibitors [38]. In our study, we observed a decrease in GPx activity at most bortezomib doses, which may prevent bortezomib resistance. GPx is critical for maintaining survival during oxidative stress [39]. Increased GPx activity can aid in maintaining the net redox state within malignant cells as a result of chemotherapy [26]. Bortezomib-resistant MM cells exhibit increased GPx activity [40,41].

A limitation of our study is the relatively small sample size, in part due to the low incidence of MM. We found spontaneous apoptosis and necrosis in cell cultures without bortezomib, which may be due to laboratory conditions, including the culture media used. In our next study, AIM-V media should be used instead of RPMI media supplemented with 10% FCS. RMPI and FCS can induce apoptosis in B cells [42]. Additionally, the present study was carried out on commercially available MM cell lines. Unfortunately, these lines do not contain all the studied *GSTT1* and *GSTM1* gene variants. Alternatively, we can attempt an antisense strategy to block the expression of *GSTT1*/*GSTM1* variants (similar to null genotypes). In addition, assessment of apoptosis could have been performed using flow cytometry. Unfortunately, this method was not available for us when we performed the experiment. Furthermore, before assessing apoptosis, we had attempted to isolate plasmocytes by magnetic method but the percentage of cells with spontaneous apoptosis was very high (>80%). Therefore, we abandoned this method and evaluated the morphology of plasmocytes using microscopy.

## 5. Conclusions

In conclusion, our data provide evidence that low-bortezomib doses decrease the activities of GST and GPx. Moreover, lower GST was associated with the null genotypes of *GSTT1* and *GSTM1* variants but only at some doses of bortezomib. Further analysis of a larger group of MM patients is recommended to confirm or negate the data we obtained.

## Figures and Tables

**Table 1:** General characteristics of the MM patients.

Variables	MM Patients, n = 80
Sex	
Male	46
Female	34
Age *	
Mean age (years)	66.95
Type of MM *	
IgG	44
IgA	16
Light chain	20
Free light chain ratio	473
Stage according to the International Staging System *	
I	20
II	16
III	44
Renal failure *	
No	54
Yes	26
Stage of kidney disease *	
G1	18
G2	18
G3A	12
G3B	14
G4	10
G5	8
Plasma cells *	
Percentage of plasma cells in bone marrow, M ± SD	31.94 ± 21.21
Cytogenetic changes	
No	68
Yes -including: del17(p13.1) t(4;14) t(14;16)	12 8 6 2
Clinical values *	
Albumins (g/dL), M ± SD	3.55 ± 0.67
β2-microglobulin (mg/L), M ± SD	6.93 ± 4.19
Calcium (mM/L), M ± SD	2.41 ± 0.33
Hemoglobin (g/dL), M ± SD	9.98 ± 1.96
Creatinine (mg/dL), M ± SD	2.10 ± 2.26
C-reactive protein (mg/L), M ± SD	10.03 ± 15.09
Chemotherapy	
Cyclophosphamide, Thalidomide, Dexamethasone (CTD)	60
Velcade, Cyclophosphamide, Dexamethasone (VCD)	18
Velcade, Thalidomide, Dexamethasone (VTD)	2
Survival	
Progression free survival (months) M ± SD	20.35 ± 16.57
Overall survival (months) M ± SD	30.77 ± 20.48

* at diagnosis.

**Table 2:** Effect of bortezomib on cell survival.

	Doses of Bortezomib	Mean Values (%)	SD	SE
Viable cells *	0 nM	89.36	7.51	1.19
	1 nM	78.75	12.28	1.94
	2 nM	75.78	12.64	2.00
	4 nM	65.92	12.78	2.02
Apoptotic cells	0 nM	5.73	5.23	0.83
	1 nM	16.00	12.28	1.94
	2 nM	16.99	6.92	1.09
	4 nM	25.03	11.12	1.76
Necrotic cells	0 nM	2.71	3.64	0.58
	1 nM	4.69	3.04	0.48
	2 nM	6.54	4.49	0.71
	4 nM	11.90	6.78	1.07

SD—standard deviation; SE—standard error; * assessed with fluorescence microscopy.

**Table 3:** Differences in the number of viable cells (%) between the control (without bortezomib, 0 nM) and bortezomib treatment groups (1 nM, 2 nM, and 4 nM).

Bortezomib Doses	Mean Difference (%)	Cohen’s d	*p*-Value
0 nM vs. 1 nM	10.61	0.92	<0.01
0 nM vs. 2 nM	13.58	1.18	<0.01
0 nM vs. 4 nM	23.44	2.04	<0.01
1 nM vs. 2 nM	2.97	-	0.68
1 nM vs. 4 nM	12.83	1.11	<0.01
2 nM vs. 4 nM	9.86	0.86	<0.01

**Table 4:** Differences in the number of apoptotic cells (%) between the control (without bortezomib, 0 nM) and bortezomib treatment groups (1 nM, 2 nM, and 4 nM).

Bortezomib Doses	Mean Difference (%)	Cohen’s d	*p*-Value
0 nM vs. 1 nM	−10.28	−1.10	<0.01
0 nM vs. 2 nM	−11.26	−1.20	<0.01
0 nM vs. 4 nM	−19.31	−2.06	<0.01
1 nM vs. 2 nM	−0.99	−0.11	0.79
1 nM vs. 4 nM	−9.03	−0.97	<0.01
2 nM vs. 4 nM	−8.04	−0.86	<0.01

**Table 5:** Differences in the number of necrotic cells (%) between the control (without bortezomib, 0 nM) and bortezomib treatment groups (1 nM, 2 nM, and 4 nM).

Bortezomib Doses	Mean Difference (%)	Cohen’s d	*p*-Value
0 nM vs. 1 nM	−1.98	−0.42	0.20
0 nM vs. 2 nM	−3.83	−0.81	<0.01
0 nM vs. 4 nM	−9.20	−1.95	<0.01
1 nM vs. 2 nM	−1.85	−0.39	0.27
1 nM vs. 4 nM	−7.22	−1.53	<0.01
2 nM vs. 4 nM	−5.37	−1.14	<0.01

**Table 6:** Differences in GSH concentration (µM/mg protein) between the control (0 nM) and bortezomib treatment groups (1 nM, 2 nM, and 4 nM).

Bortezomib Doses	Mean Difference (µM/mg Protein)	Cohen’s d	*p*-Value
0 nM vs. 1 nM	0.04	-	0.90
0 nM vs. 2 nM	0.04	-	0.91
0 nM vs. 4 nM	0.07	0.53	0.05
1 nM vs. 2 nM	0.01	-	1.00
1 nM vs. 4 nM	0.03	-	1.00
2 nM vs. 4 nM	0.03	-	1.00

**Table 7:** Differences in GST activity (U·mg^−1^ protein) between the control (0 nM) and bortezomib treatment groups (1 nM, 2 nM, and 4 nM).

Bortezomib Doses	Mean Difference (U·mg^−1^ Protein)	Cohen’s d	*p*-Value
0 nM vs. 1 nM	0.01	0.09	<0.01
0 nM vs. 2 nM	0.03	0.24	<0.01
0 nM vs. 4 nM	0.05	0.44	<0.01
1 nM vs. 2 nM	0.02	0.15	<0.01
1 nM vs. 4 nM	0.04	0.35	<0.01
2 nM vs. 4 nM	0.02	0.20	<0.01

**Table 8:** Differences in GR activity (U·mg^−1^ protein) between the control (0 nM) and bortezomib treatment groups (1 nM, 2 nM, and 4 nM).

Bortezomib Doses	Mean Difference (U·mg^−1^ Protein)	Cohen’s d	*p*-Value
0 nM vs. 1 nM	−0.02	-	1.00
0 nM vs. 2 nM	−0.04	-	1.00
0 nM vs. 4 nM	−0.13	−0.64	0.02
1 nM vs. 2 nM	−0.02	-	1.00
1 nM vs. 4 nM	−0.11	−0.55	0.06
2 nM vs. 4 nM	−0.09	-	0.26

**Table 9:** Differences in GPx activity (U·mg^−1^ protein) between the control (0 nM) and bortezomib treatment groups (1 nM, 2 nM, and 4 nM).

Bortezomib Doses	Mean Difference (U·mg^−1^ Protein)	Cohen’s d	*p*-Value
0 nM vs.1 nM	0.02	0.16	0.96
0 nM vs. 2 nM	0.05	0.43	<0.01
0 nM vs. 4 nM	0.10	0.83	<0.01
1 nM vs. 2 nM	0.03	0.26	0.15
1 nM vs. 4 nM	0.08	0.67	<0.01
2 nM vs. 4 nM	0.05	0.40	<0.01

**Table 10:** Hardy-Weinberg equilibrium for *GST* polymorphisms in the case and control groups according to expected (E) and observed (O) values.

	Null (Homozygotes)	Present (Heterozygotes)	Present (Homozygotes)	Total	HWE *p*-Value and χ^2^ *
CONTROL	*GSTT1*				
E	31.9	49.2	18.9	100	*p* = 0.97, χ^2^ = 0.001
O	32	49	19	100	
CASE					
E	24.2	39.6	16.2	80	*p* = 0.92, χ^2^ = 0.008
O	24	40	16	80	
CONTROL	*GSTM1*				
E	39.7	46.6	13.7	100	*p* = 0.89, χ^2^ = 0.017
O	40	46	14	100	
CASE					
E	24.2	39.6	16.2	80	*p* = 0.92, χ^2^ = 0.008
O	24	40	16	80	

* if χ^2^ ≤ 3.84 and the corresponding *p* ≥ 0.05, then the population is in HWE.

**Table 11:** Comparison of *GST* polymorphisms impact on the MM risk.

Genotypes	MM Patients n = 80	Controls n = 100	OR	95%CI	*p*-Value
*GSTT1*					
present	56	68	referent	-	-
null	24	32	1.01	0.58–2.08	0.77
*GSTM1*					
present	56	60	referent	-	-
null	24	40	1.55	0.83–2.09	0.16

**Table 12:** The combined effect of *GST* polymorphisms on the MM risk.

*GSTT1*	*GSTM1*	MM Patients n	Controls n	OR	95%CI	p-Value
present	present	42	46	R	-	-
null	present	14	14	0.91	0.39–2.14	0.83
present	null	14	22	1.43	0.65–3.16	0.37
null	null	10	18	1.64	0.68–3.95	0.26

## Data Availability

The clinical data used to support the findings of this study are available from the corresponding author upon request.
